# A Strong Machine Learning Classifier and Decision Stumps Based Hybrid AdaBoost Classification Algorithm for Cognitive Radios †

**DOI:** 10.3390/s19235077

**Published:** 2019-11-20

**Authors:** Siji Chen, Bin Shen, Xin Wang, Sang-Jo Yoo

**Affiliations:** 1 School of Communication and Information Engineering (SCIE), Chongqing University of Posts and Telecommunications (CQUPT), Chongqing 400-065, China; cqchensj@outlook.com (S.C.); s170101160@stu.cqupt.edu.cn (X.W.); 2 Department of Information and Communication Engineering, Inha University, Incheon 402-751, Korea; sjyoo@inha.ac.kr

**Keywords:** machine learning, classifier, decision stump, AdaBoost, energy vector, cooperative spectrum sensing, cognitive radio network (CRN)

## Abstract

Machine learning (ML) based classification methods have been viewed as one kind of alternative solution for cooperative spectrum sensing (CSS) in recent years. In this paper, ML techniques based CSS algorithms are investigated for cognitive radio networks (CRN). Specifically, a strong machine learning classifier (MLC) and decision stumps (DS) based adaptive boosting (AdaBoost) classification mechanism is proposed for pattern classification of the primary user’s behavior in the network. The conventional AdaBoost algorithm only combines multiple sub-classifiers and produces a strong weight based on their weights in classification. Taking into account the fact that the strong MLC and the weak DS serve as different sub-classifiers in classification, we propose employing a strong MLC as the first-stage classifier and DS as the second-stage classifiers, to eventually determine the class that the spectrum energy vector belongs to. We verify in simulations that the proposed hybrid AdaBoost algorithms are capable of achieving a higher detection probability than the conventional ML based spectrum sensing algorithms and the conventional hard fusion based CSS schemes.

## 1. Introduction

Cognitive radio (CR) is commonly viewed as a promising technology that tremendously alleviates the increasing pressure on current rigid spectrum resource allocation regimes, by enabling dynamic access to the licensed spectrum in an opportunistic manner [1]. The opportunistic spectrum acquisition capability of CR systems generally relies on the spectrum sensing technologies that they adopt to identify the licensed spectrum status [2,3]. Cognitive users, also known as secondary users (SUs), are permitted to utilize the licensed spectrum only if they can assure themselves that the licensed spectrum is not temporarily occupied by the primary users (PUs). Generally, single user based spectrum sensing technologies can be categorized into blind and knowledge aided approaches [4]. Among various spectrum sensing methods, energy detection (ED) is an extensively used technique that requires no a priori knowledge of the PU signal and the channel environment. Capturing the received signal energy within a certain frequency band, a simple threshold test could agilely indicate whether the PU signal exists in the licensed spectrum or not. However, it is well known that the easy-to-implement energy detectors are vulnerable to noise power uncertainty effects and could not perform well under low signal to noise ratio (SNR) conditions, especially in severe multipath fading and shadowing environments.

In order to tackle the problem of single user based spectrum sensing, cooperative spectrum sensing (CSS) has been widely investigated, where multiple SUs collaborate to make a global decision on the licensed spectrum status. During each sensing interval, all SUs will report their spectrum observations or decisions to the fusion center (FC) and subsequently the FC makes a final decision based on its predefined decision strategy or fusion criterion [5]. Typical hard decision fusion based CSS schemes can be found in [6,7], in which logical operations are proposed for fusing the individual decisions collected from the cooperative SUs in the network.

Apart from the conventional ED based spectrum sensing schemes, machine learning (ML) based classification methods have been attracting attention in recent years, where the problem of spectrum status identification is solved by classifying the collected spectrum observations as the PU signal component contained data or the noise-only data [8,9,10]. For the purpose of spectrum sensing, ML approaches are classifiers to improve the detection performance of CSS and spectrum prediction, which is a part of spectrum sharing approaches [11]. ML based classifiers have the function of automatic learning over a large amount of training data and are hence able to make spectrum status predictions on the test data, which in some general way allays the nuisance effects of multipath fading and shadow, encountered by threshold-test based traditional spectrum sensing strategies. Therefore, the ML based classifiers usually perform better than energy detectors and the logical criteria based CSS schemes. As for the implementation of ML algorithms, they can be roughly divided into two types: unsupervised learning, e.g., K-means, and supervised learning, e.g., support vector machine (SVM) and K-nearest neighbors (KNN) [12,13,14]. The unsupervised methods directly model the input training data without exactly known labels for each training data point. In other words, they can not be sure whether the classification result of the training samples is correct or not [15]. The feature of these algorithms is beneficial in that they demand no a priori knowledge of the PU behavior and only rely on the training samples to automatically find their potential labels according to the training samples themselves.

Unlike unsupervised learning, the supervised learning methods analyze the relationship between the training data and the corresponding labels readily available for them, and map the input test data to the appropriate prediction or decision. Specifically, when the training phase is completed, the generalized model is applied to the new test data and their labels are thus accordingly predicted. Compared with unsupervised learning, the supervised learning methods are characterized by demanding more a priori knowledge from the input training samples and the corresponding true labels.

As a typical classifier, SVM is studied in [16] for CSS in detail, with an aim to find a linearly separable hyperplane with the help of support vectors by maximizing the margin of the classifier while minimizing the sum of classification errors. In [17,18], KNN is introduced and proved to be not only one of the simplest machine learning algorithms, but also one of the most basic, and best text categorization algorithms in case-based learning methods. However, to correctly apply KNN, we need to choose an appropriate value for *K*, because the success of classification is highly dependent on this value. In this sense, it may be concluded that the KNN method is biased by *K*, and there are many ways of choosing the *K* value, among which a simple method is to run the algorithm many times with different *K* values and choose the one with the best performance [19]. However, this might not be a satisfactory plug-and-play solution for spectrum sensing in practice. Essentially as a most well-known clustering algorithm, the K-means algorithm clusters the data points with high similarity into the same cluster according to the principle of similarity [20]. Due to its simplicity and efficiency, it has become the most widely used clustering algorithm [21]. Since different classifiers have various advantages and drawbacks, combining different individual classifiers into a comprehensive one with better performance is a straightforward solution. In [22,23], the adaptive boosting (AdaBoost) algorithm is introduced, for which the constructed classifier is composed of multiple weaker models that are independently trained and whose predictions are combined to make the overall prediction.

Noticing that employing ML techniques for CSS may be currently a novel solution in discerning the licensed spectrum status, we study the potentiality and feasibility of adopting ML algorithms in CSS, where the FC handles the spectrum data in a totally different manner compared to the conventional hard decision fusion based CSS schemes. To be more specific, we propose a hybrid AdaBoost classification algorithm which combines a strong ML algorithm (e.g., SVM, K-Means, and KNN) and multiple weak ML classifier (e.g., DS) for CSS in the cognitive radio network (CRN). Taking into account the fact that the ML classifier and DS serve as relatively strong and weak sub-classifiers respectively, the proposed algorithm employs one of the common ML classification algorithms (SVM, KNN, and K-Means) as the first-stage classifier and DS as the second-stage classifier to eventually determine the class that the spectrum observations belong to. For the scenario that there is one single PU and multiple SUs in the CRN, the proposed hybrid AdaBoost classification algorithm yields higher detection probability than the pure ML classification algorithms themselves and the DS based AdaBoost classification algorithm as well. In our proposed schemes, the SVM based hybrid AdaBoost performs the best. When compared with the traditional CSS schemes, e.g., AND and OR criteria based hard decision fusion algorithms, the proposed hybrid AdaBoost classification algorithm achieves better performance too. All these verifications prove that the proposed algorithms could be utilized in practice as a newfangled solution for CSS.

The remainder of this paper is organized as follows. In Section 2, we present the system model and some assumptions. Section 3 introduces the conventional spectrum sensing methods. Section 4 investigates various typical ML classifiers for CSS. In Section 5, we propose and describe the hybrid AdaBoost classification algorithm in detail. In Section 6, performance evaluation results of the proposed hybrid AdaBoost classification algorithm are given and compared with the conventional algorithms. Finally, Section 7 concludes the paper.

## 2. System Model

We consider a CRN consisting of a single PU transmitter and *N* SU receivers indexed by n=1,2,⋯,N. In the CRN, the *n*-th SU is located at the geographic coordinate $C_{\mathrm{SU}}^{n}={\left[x_{n},y_{n}\right]}^{T}$. We assume that the probability of the PU being active over the licensed spectrum is $P_{\mathrm{ON}}$ and there are two hypotheses, $H_{0}$ and $H_{1}$ with probability $\left(1-P_{\mathrm{ON}}\right)$ and $P_{\mathrm{ON}}$, respectively.

The received signal at the *n*-th SU is expressed as

(1) $$y_{n}\left(i\right)=\left\{\begin{array}{cc} r_{n}\left(i\right) & \;H_{0},\\ g_{n}\sqrt{P_{\mathrm{tx}}}x\left(i\right)+r_{n}\left(i\right) & \;H_{1}, \end{array}\right.$$

where $g_{n}$ denotes the channel gain from the PU transmitter to *n*-th SU, x(i) is the PU’s transmitted signal, $P_{\mathrm{tx}}$ is the PU’s transmitting power, and $r_{n}\left(i\right)$ is the additive white gaussian noise (AWGN) with zero-mean and variance $\eta =E\left[|r_{n}\left(i\right){|}^{2}\right]=\sigma_{0}^{2}$. If the time duration of each spectrum sensing interval is denoted as τ and the spectrum bandwidth is *W*, each SU usually grasps 2Wτ samples of its received signal during the sensing duration τ.

The noise variance normalized energy statistic of the *n*-th SU in the first CSS phase (It is assumed that multiple SUs collect their spectrum observations in the first phase of CSS and the second phase is for global decision making in the FC.) can be denoted by $Y_{n}$:

(2) $$Y_{n}=\frac{1}{\eta }\;\sum_{i=1}^{2W\tau }{\left|y_{n}\left(i\right)\right|}^{2}.$$

In the second CSS phase, all SUs report their spectrum decision $D_{n}$ or energy observation $Y_{n}$ to the FC as:

(3) $$\begin{array}{cc} D & ={\left[D_{1},D_{2},\cdots ,D_{N}\right]}^{T},\\ Y & ={\left[Y_{1},Y_{2},\cdots ,Y_{N}\right]}^{T}, \end{array}$$

where the FC operates over D or Y via different criteria to make the global decision on the spectrum status.

The power attenuations between the PU and the SU depend on the channel coefficient $g_{n}$ in that $G_{n}={\left|g_{n}\right|}^{2}$, where the power attenuation coefficient $G_{n}$ can be expressed as:

(4) $$G_{n}=PL\left({∥C_{\mathrm{PU}}-C_{\mathrm{SU}}^{n}∥}_{2}\right)\cdot \psi_{n}\cdot v_{n},$$

where ${∥\cdot ∥}_{2}$ stands for the Euclidean distance or equivalently the L2-norm of the vector $C_{\mathrm{PU}}-C_{\mathrm{SU}}^{n}$, $PL\left(d\right)=d^{-\rho }$ is the pathloss for the relative distance *d* with the propagation loss exponent ρ, $C_{\mathrm{PU}}={\left[x_{\mathrm{PU}},y_{\mathrm{PU}}\right]}^{T}$ denotes the coordinate of the PU tranmitter, $\psi_{n}$ is the shadow fading coefficient, and $v_{n}$ the multipath fading coefficient.

## 3. Conventional Spectrum Sensing Methods

### 3.1. Single User Based Energy Detection

According to the well-known ED, the *n*-th SU measures the strength of the received signal $y_{n}$ and obtains the test statistic $Y_{n}$ and the spectrum decision $D_{n}$ via the threshold-test:

(5) $$\begin{array}{c} Y_{n}≷\lambda_{n}\begin{array}{cc} ↗\; & D_{n}=1,\\ ↘\; & D_{n}=0, \end{array} \end{array}$$

where $\lambda_{n}$ is the pre-calibrated threshold, depending on the desired false alarm probability under the criterion of constant false alarm probability (CFAP), and the binary decision $D_{n}=0$ and $D_{n}=1$ indicates the cases of PUs being idle and active in the CRN, respectively.

### 3.2. Multi-User Based Hard Decision Fusion

On obtaining the spectrum decisions $D_{n}$, the *n*-th SU may cooperate with multiple SUs in its neighborhood to strengthen the sensing reliability through the hard decision fusion criteria, e.g., AND, OR, and Vote, as:

(6) $$\begin{array}{c} \Lambda_{n}=\sum_{t\in Q_{n},t\neq n}D_{t}+D_{n}≷\lambda_{T}\begin{array}{cc} ↗\; & {\tilde{D}}_{n}={\hat{H}}_{1},\\ ↘\; & {\tilde{D}}_{n}={\hat{H}}_{0}, \end{array} \end{array}$$

where $D_{t}$ is the spectrum decision of the *t*-th SU, $Q_{n}$ is the index set of the *n*-th SU’s neighbouring SU with the set cardinality of $|Q_{n}|$, and $\lambda_{T}$ is an integer threshold for the hard decision fusion schemes. The hard decision fusion scheme described in Equation (6) may boil down to the AND scheme for $\lambda_{T}=1$, the OR scheme for $\lambda_{T}=\left|Q_{n}\right|+1$, and the $\lambda_{T,0}$-out-of-$\left(|Q_{n}|+1\right)$ (Vote) scheme for $\lambda_{T}=\lambda_{T,0}$, respectively. Decisions ${\hat{H}}_{0}$ and ${\hat{H}}_{1}$ are actually the final decisions for the *n*-th SU indicating the cases of PUs being idle and active in the CRN, respectively.

The spectrum sensing performance is usually evaluated in terms of detection probability $P_{D}=Pr\left({\hat{H}}_{1}\;|\;H_{1}\right)$ and the false alarm probability $P_{\mathrm{FA}}=Pr\left({\hat{H}}_{1}\;|\;H_{0}\right)$. The detection probability $P_{D}$ is a function of the SNR and the $P_{\mathrm{FA}}$ is usually predetermined as the desired performance for spectrum sensing.

## 4. Typical ML Classifiers for CSS

In this section, we investigate and propose some typical ML classifiers for the proposed CSS model. For ML method based CSS, a sufficiently large number of training energy vectors are demanded as training samples to train the classifiers. Let $x^{\left(l\right)}={\left[X_{1}^{\left(l\right)},X_{2}^{\left(l\right)},\cdots ,X_{N}^{\left(l\right)}\right]}^{T}$ denote the *l*-th training energy vector obtained as $X_{n}=\frac{1}{\eta }\;\sum_{i=1}^{2W\tau }{\left|y_{n}\left(i\right)\right|}^{2}$ and $c^{\left(l\right)}$ the spectrum availability flag (or label) corresponding to $x^{\left(l\right)}$. Thus, the training set of energy vectors are $\bar{X}=\left\{x^{\left(1\right)},x^{\left(2\right)},\cdots ,x^{\left(L\right)}\right\}$ and the spectrum availability labels are represented by $\bar{c}=\left\{c^{\left(1\right)},c^{\left(2\right)},\cdots ,c^{\left(L\right)}\right\}$, where *L* is the number of training samples (energy vectors) in the training set. After the classifier is successfully trained over the given training set $\bar{X}$, it is able to classify the input test energy vector $x^{*(m)}$ with label $c^{*(m)},$ denoting its true spectrum availability label. In addition, the set of test energy vectors can be denoted as $X^{*}=\left\{x^{*(1)},x^{*(2)},\cdots ,x^{*(M)}\right\}$ and the set of corresponding spectrum availability labels is $c^{*}=\left\{c^{*(1)},c^{*(2)},\cdots ,c^{*(M)}\right\}$, where *M* is the number of test samples in the test set. If ${\hat{c}}^{\left(m\right)}$ is used to denote the *m*-th test sample’s spectrum availability label decided by the classifier, it is either the spectrum available label (i.e., ${\hat{c}}^{\left(m\right)}=-1$) or the spectrum unavailable label (i.e., ${\hat{c}}^{\left(m\right)}=1$). If the test energy vector $y^{*(m)}$ is classified and labeled as spectrum available, it means that there is no PU in the active state and the licensed spectrum is available for the SU to access; otherwise, the SU cannot gain spectrum opportunity when the label is drawn as spectrum unavailable.

Therefore, the spectrum availability is correctly determined in the case that ${\hat{c}}^{\left(m\right)}=c^{*(m)}$, while mis-detection (or false alarm) occurs in the case that ${\hat{c}}^{\left(m\right)}=-1$ and $c^{*(m)}=1$ (or ${\hat{c}}^{\left(m\right)}=1$ and $c^{*(m)}=-1$). Thus, the prediction error ($P_{e}$) is defined as the probability of mis-prediction of the status of the licensed spectrum:

(7) $$P_{e}=\lim_{M\to \infty }\frac{1}{M}\sum_{m=1}^{M}I({\hat{c}}^{\left(m\right)}\neq c^{*(m)}),$$

where I(.) is the indicator function that takes the value 1 if its argument is true and 0 otherwise.

### 4.1. K-Means Clustering

The K-means clustering algorithm operates in an unsupervised manner and partitions a set of the training energy vectors (i.e., $\bar{X}=\left\{x^{\left(1\right)},x^{\left(2\right)},\cdots ,x^{\left(L\right)}\right\}$) into *K* disjoint clusters, which represents the *K* categories of training vectors. In other words, the training energy vectors can be assigned into *K* subsets of spectrum observations denoted by cluster labels $C_{k}$, k=1,2,⋯,K. Normally, if we purposely set the number of clusters as two in advance, we can obtain two clusters by using the K-means algorithm without knowing the true labels that these two clusters actually correspond to. In spectrum sensing, it is applicable to set cluster 1 and cluster 2 as the cluster with channel availability label -1 and the cluster with channel availability label 1, respectively. In this way, the K-means clustering algorithm serves as an ML classifier, equivalently. The core idea of K-means is each cluster has a centroid that is defined as the mean of all training energy vectors within itself. Let $\mu_{k}$ be the centroid of cluster *k* and $\gamma_{lk}\in \left\{0,1\right\}$ be the indicative variable. We define $\gamma_{lk}=1$, if the energy vector $x^{\left(l\right)}$ is assigned to cluster *k*, otherwise, $\gamma_{lk}=0$. Through several iterations of distance calculation, the values of $\mu_{k}$ are updated sample by sample as follows:

(8) $$\mu_{k}=\frac{\sum_{l=1}^{L}\gamma_{lk}x^{\left(l\right)}}{\sum_{l=1}^{L}\gamma_{lk}},$$

The objective function of K-means is to minimize the squared error in clustering:

(9) $$J\left(\gamma ,\bar{\mu },\bar{X}\right)=\sum_{l=1}^{L}\sum_{k=1}^{K}\gamma_{lk}{∥x^{\left(l\right)}-\mu_{k}∥}_{2}^{2},$$

where $\gamma ={\left[\gamma_{11},\gamma_{21},\cdots ,\gamma_{L1},\gamma_{12},\cdots ,\gamma_{L2},\cdots \gamma_{LK}\right]}^{T}$ and $\bar{\mu }={\left[\mu_{1},\mu_{2},\cdots ,\mu_{K}\right]}^{T}$.

**Figure 1:**
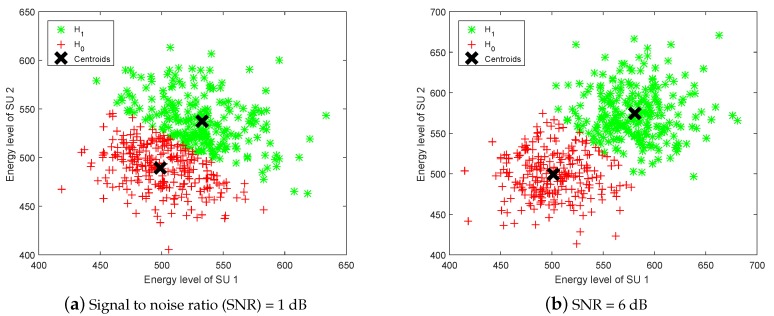
Scatter plot of energy vectors classified by K-means.

Figure 1 shows the two-user based classification results of the K-means clustering algorithm on the training samples when SNR =1 dB and SNR =6 dB respectively. We can see the farther the distance between samples belonging to two different labels, the better the classification result that can be obtained.

### 4.2. Support Vector Machine

**Figure 2:**
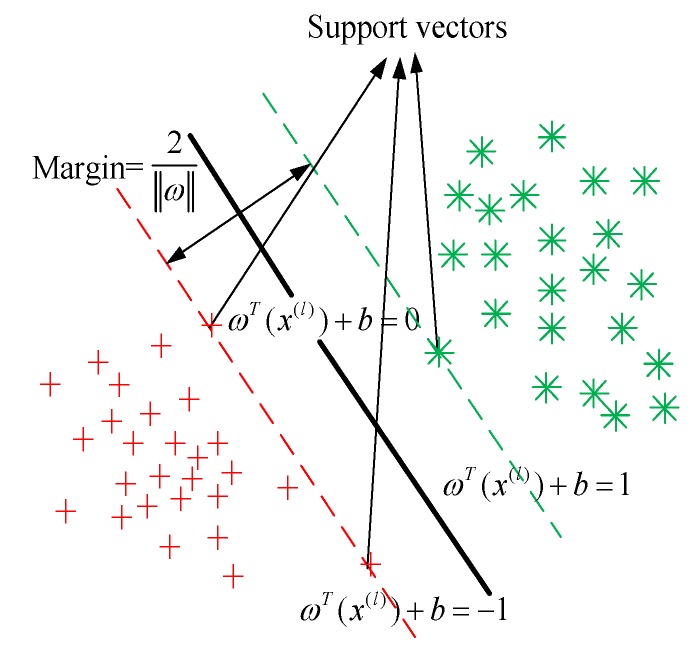
Support vector machine (SVM) model.

The SVM provides a binary model in machine learning which strives to find a linearly separable hyperplane with the help of support vectors (i.e., energy vectors that lie closest to the decision surface) by maximizing the margin of the classifier while minimizing the sum of classification errors [24]. As shown in Figure 2, the learning strategy of SVM is to maximize the margin and its learning goal is to find a hyperplane in the *N*-dimensional sample space.

The hyperplane equation can be expressed as

(10) $$\omega^{T}x^{\left(l\right)}+b=0,$$

where ω is the weighting vector and *b* is the bias. Based on ω, we need to minimize the vector norm of ω so as to maximize the category margin, and hence the objective function is

(11) $$\begin{array}{c} \mathrm{Min}\;\frac{1}{2}{∥\omega ∥}^{2}\\ s.t.\;c^{\left(l\right)}\left(\omega^{T}x^{\left(l\right)}+b\right)\geq 1. \end{array}$$

Therefore, the SVM should satisfy the following condition for all l∈{1,2,⋯,L}:

(12) $$c^{\left(l\right)}=\left\{\begin{array}{cc} 1, & \mathrm{if}\;\omega^{T}x^{\left(l\right)}+b\geq 1,\\ -1, & \mathrm{if}\;\omega^{T}x^{\left(l\right)}+b\leq -1. \end{array}\right.$$

In practice, when a test energy vector $x^{*(m)}$ is fed into the SVM model, the SVM can determine which class it belongs to through the following rules:

(13) $${\hat{c}}^{\left(m\right)}=\left\{\begin{array}{cc} 1, & \mathrm{if}\;\omega^{T}x^{*(m)}+b\geq 1,\\ -1, & \mathrm{if}\;\omega^{T}x^{*(m)}+b\leq -1. \end{array}\right.$$

However, in practice for most of the time, the test samples are usually not linearly separable. For this case, the hyperplane satisfying such conditions does not exist at all. Then, we need to find a fixed nonlinear feature mapping function ϕ to map the non-linear samples into a new feature space and use a linear SVM in the feature space [25]. Hence, the non-linear SVM should satisfy the following condition for all l∈{1,2,⋯,L}:

(14) $$c^{\left(l\right)}=\left\{\begin{array}{cc} 1, & \mathrm{if}\;\omega^{T}\phi \left(x^{\left(l\right)}\right)+b\geq 1,\\ -1, & \mathrm{if}\;\omega^{T}\phi \left(x^{\left(l\right)}\right)+b\leq -1, \end{array}\right.$$

and the decision rule for the non-linear SVM is given as:

(15) $${\hat{c}}^{\left(m\right)}=\left\{\begin{array}{cc} 1, & \mathrm{if}\;\omega^{T}\phi \left(x^{*(m)}\right)+b\geq 1,\\ -1, & \mathrm{if}\;\omega^{T}\phi \left(x^{*(m)}\right)+b\leq -1. \end{array}\right.$$

Although the training energy vectors have been mapped into a higher dimensional feature space, practically we cannot achieve a perfect linearly separable hyperplane that satisfies the condition in Equation (15) for each $x^{\left(l\right)}$. Hence, we rewrite the optimization problem as a convex optimization problem as follows:

(16) $$\begin{array}{c} \mathrm{Min}\;\frac{1}{2}{∥\omega ∥}^{2}+C\sum_{l=1}^{L}\xi^{\left(l\right)}\\ s.t.\;c^{\left(l\right)}\left(\omega^{T}x^{\left(l\right)}+b\right)\geq 1-\xi^{\left(l\right)},\;l=1,2,\cdots ,L\\ \;\;\;\;\;\;\;\;\;\xi^{\left(l\right)}\geq 0,l=1,2,\cdots ,L, \end{array}$$

where *C* is the soft margin constant, for which a larger *C* means the assignment of a higher penalty to errors, and $\xi^{\left(l\right)}$ is the slack variable.

**Figure 3:**
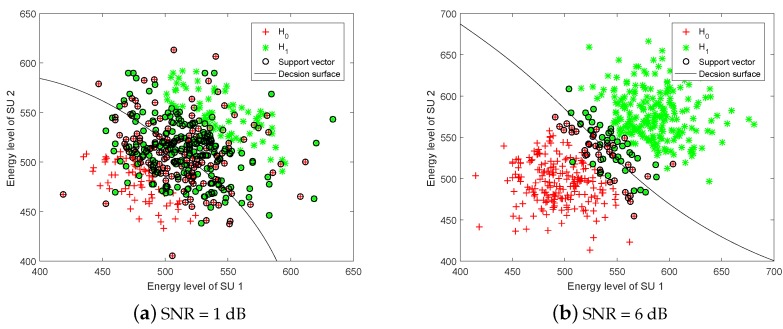
Scatter plot of energy vectors classified by SVM.

Figure 3 shows the training samples classified by SVM-rbf. We can notice that the decision surface divides the energy vectors in each class as clearly as possible, which leads to improved detection performance. Also, the decision surface can separate energy vectors more accurately as the SNR increases.

### 4.3. K-Nearest-Neighbor

The supervised K-nearest neighbors is a case-based learning method, which keeps all the training samples for classification. Being a lazy learning method prohibits it in many applications such as dynamic web mining for a large repository, as the KNN classifier requires storing the whole training set and may be too costly when this set is too large. One way to improve its efficiency is to find some representatives of the whole training data for classification, building an inductive learning model from the training samples, and classifying the test samples based on a similarity measure (e.g., distance functions), for example:

(17) $$\mathrm{Euclidean}:{∥x^{*(m)}-x^{\left(l\right)}∥}_{2}=\sqrt{\sum_{n=1}^{N}{\left|x_{n}^{*(m)}-x_{n}^{\left(l\right)}\right|}^{2}},\;\;l=1,2,\cdots ,L,$$

(18) $$\mathrm{Manhattan}:{∥x^{*(m)}-x^{\left(l\right)}∥}_{1}=\sum_{n=1}^{N}\left|x_{n}^{*(m)}-x_{n}^{\left(l\right)}\right|,\;\;l=1,2,\cdots ,L.$$

According to the given distance measure, *K* samples with the nearest neighbor of test energy vector $x^{*(m)}$ are found in the training set $\bar{x}$ and the domain of covering these *K* samples is $N_{K}$. Among them, the setting of *K* value is generally lower than the square root of the number of samples, and it must be an odd number. Then the spectrum availability label for the spectrum data $x^{*(m)}$ can be predicted based on classification decision rules (e.g., majority voting) as:

(19) $${\hat{c}}^{\left(m\right)}=\underset{c^{\left(k\right)}}{\arg\max}\sum_{k=1}^{K}I\left(c^{\left(k\right)}=\chi \right),$$

where $\chi \in \{c^{\left(1\right)},c^{\left(2\right)},\cdots ,c^{\left(L\right)}\}$ and $c^{\left(k\right)}$ is the training label of the *k*-th nearest neighbor in $N_{K}$.

### 4.4. AdaBoost Algorithm

As for the weak classifier DS, it is a one-dimension decision tree, that uses only a single attribute of the spectrum data for splitting and makes only one judgment on each attribute (i.e., one of the test sample’s dimensions). It is also well known that the decision stumps (DS) are often used as sub-classifiers in ensemble methods.

**Figure 4:**
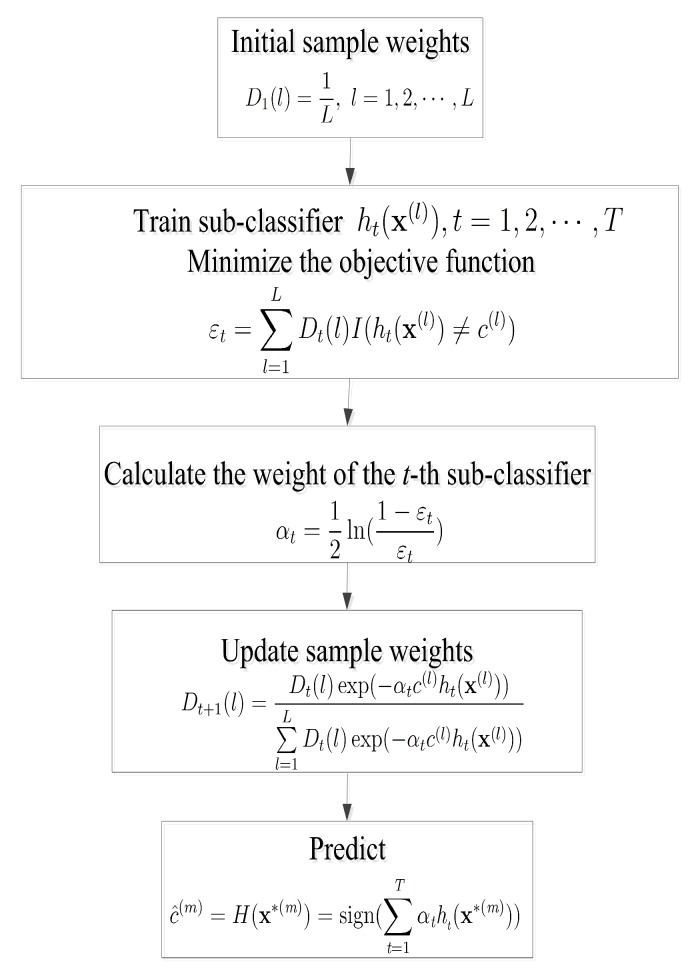
Flowchart of the adaptive boosting (AdaBoost) classification algorithm.

In addition to the weak DS and strong ML classifiers, AdaBoost (adaptive boosting) algorithm is an ensemble learning algorithm composed of plenty of sub-classifiers to overcome the drawbacks of poor classification of individual sub-classifiers [26,27]. The core idea is to train different sub-classifiers $h_{t},t\in \left\{1,2,\cdots ,T\right\}$ on the same training samples. As one kind of weak classifier, the DS is often used as sub-classifier in the AdaBoost algorithm [28,29], and these weak sub-classifiers are grouped together to construct a final stronger classifier. In Figure 4, we present the flowchart of the AdaBoost algorithm.

As a combination of multiple sub-classifiers, the AdaBoost algorithm can be used for classification by choosing suitable sub-classifiers. The conventional AdaBoost algorithm adopts DS as sub-classifiers and it is proved to be efficient for many applications. However, complexity of this pure DS based structure is O(LT), and clearly it would spend more time to train and predict with the increase of the number of training samples and iteration rounds.

## 5. Hybrid AdaBoost Classification Algorithm

In this section, we propose a hybrid AdaBoost algorithm which uses a strong ML algorithm and multiple DS as sub-classifiers. The philosophy of employing both the strong ML algorithm and the DS lies in the feature of non-misclassification on training samples of decision stumps based AdaBoost algorithm and the feature of high classification accuracy of the strong ML algorithm. The hybrid AdaBoost algorithm adopts a strong ML algorithm to be a first sub-classifier and it is supposed to obtain the same classification result as that of many DS classifiers jointly operating together. Therefore, it decreases the number of iteration rounds and demands lower computational complexity than the conventional AdaBoost algorithm. As expected and verified, the proposed hybrid structure of the sub-classifiers in AdaBoost helps increase the detection probability and reduces the time consumed in operations. In our work, the aforementioned typical ML based strong classifiers (e.g., SVM and KNN) and clustering algorithm (e.g., K-Means) are first considered for serving as sub-classifiers in constructing a comprehensive classification algorithm to achieve better performance. We must point out that only cluster 1 or cluster 2 are obtained by K-Means and are not able to get the labels of data. But K-Means can still be used here in AdaBoost as the first sub-classifier, as long as we set cluster 1 and cluster 2 as label −1 and label 1 respectively.

To be more specific, we assign the initial weight $D_{1}$ to each training sample as 1/L in the first step. A strong ML classifier is adopted as the first sub-classifier and multiple DS are used as the following sub-classifiers (i.e., $h_{t}=h_{\mathrm{DS}},t=2,3,\cdots ,T$). After hybrid AdaBoost classification is performed, we can obtain the weight of each sub-classifier as follows:

(20) $$\alpha_{t}=\frac{1}{2}\ln\left(\frac{1-\varepsilon_{t}}{\varepsilon_{t}}\right),$$

where $\varepsilon_{t}$ is the classification error at the *t*-th AdaBoost round by using the *t*-th sub-classifier and it is defined as:

(21) $$\varepsilon_{t}≜\sum_{l=1}^{L}D_{t}\left(l\right)I\left(h_{t}\left(x^{\left(l\right)}\right)\neq c^{\left(l\right)}\right),$$

where $h_{t}\left(x^{\left(l\right)}\right)$ is the predicted class of $x^{\left(l\right)}$ by classifier $h_{t}$ and $D_{t}\left(l\right)$ denotes the *l*-th training sample’s weight at the *t*-th AdaBoost round.

In the proposed hybrid AdaBoost algorithm, the correctly classified sample’s weight increases and the falsely classified sample’s weight decreases at the end of each AdaBoost round, with the weights updated as:

(22) $$D_{t+1}\left(l\right)=\frac{D_{t}\left(l\right)\exp\left(-\alpha_{t}c^{\left(l\right)}h_{t}\left(x^{\left(l\right)}\right)\right)}{Z_{t}},$$

where $Z_{t}$ presents the normalization factor which is defined as:

(23) $$Z_{t}≜\sum_{l=1}^{L}D_{t}\left(l\right)\exp\left(-\alpha_{t}c^{\left(l\right)}h_{t}\left(x^{\left(l\right)}\right)\right).$$

Since a strong ML classifier’s classification performance is generally better than that of the single DS, the weight of the strong ML classifier $\alpha_{1}$ classifier is usually greater than that of the other single DS classifiers $\left\{\alpha_{2},\alpha_{3},\cdots ,\alpha_{T}\right\}$. Finally, these sub-classifiers are linearly combined to obtain a final classifier to predict the spectrum availability label on the basis of the test samples $x^{*(m)}$. The classification result for the CSS is then obtained as:

(24) $${\hat{c}}^{\left(m\right)}=H\left(x^{*(m)}\right)=sign\left(\sum_{t=1}^{T}\alpha_{t}h_{t}\left(x^{*(m)}\right)\right),$$

where H(.) represents the hybrid AdaBoost operator.

Specifically, the proposed AdaBoost algorithm based on the hybrid structure of a strong ML classifier and multiple DS is described in Algorithm 1.

**Algorithm 1** A Strong Machine Learning Classifier and Decision Stumps Based Hybrid Adaboost Classification Algorithm.**Require:**$\bar{X},\;\left\{c^{\left(1\right)},c^{\left(2\right)},\cdots ,c^{\left(L\right)}\right\}$,$\;\;X^{*}$,T(number of sub-classifiers). **Ensure:**${\hat{c}}^{\left(m\right)},m\in 1,2,\cdots ,M$.
1:Initialize sample weight   $D_{1}\left(l\right)=\frac{1}{L}$2:$\varepsilon_{1}=\sum_{l=1}^{L}D_{1}\left(l\right)I\left(h_{1}\left(x^{\left(l\right)}\right)\neq c^{\left(l\right)}\right)$3:$\alpha_{1}=\frac{1}{2}\ln\left(\frac{1-\varepsilon_{1}}{\varepsilon_{1}}\right)$4:$D_{2}\left(l\right)=\frac{D_{1}\left(l\right)\exp\left(-\alpha_{1}c^{\left(l\right)}h_{1}\left(x^{\left(l\right)}\right)\right)}{Z_{1}}$5:**for**t=2toT**do**6:    $\varepsilon_{t}=\sum_{l=1}^{L}D_{t}\left(l\right)I\left(h_{t}\left(x^{\left(l\right)}\right)\neq c^{\left(l\right)}\right)$7:       $\alpha_{t}=\frac{1}{2}\ln\left(\frac{1-\varepsilon_{t}}{\varepsilon_{t}}\right)$8:          $D_{t+1}\left(l\right)=\frac{D_{t}\left(l\right)\exp\left(-\alpha_{t}c^{\left(l\right)}h_{t}\left(x^{\left(l\right)}\right)\right)}{Z_{t}}$9:**end for**10:Predict the class of the *m*-th test sample   ${\hat{c}}^{\left(m\right)}=sign\left(\sum_{t=1}^{T}\alpha_{t}h_{t}\left(x^{*(m)}\right)\right)$

## 6. Simulations Results

**Figure 5:**
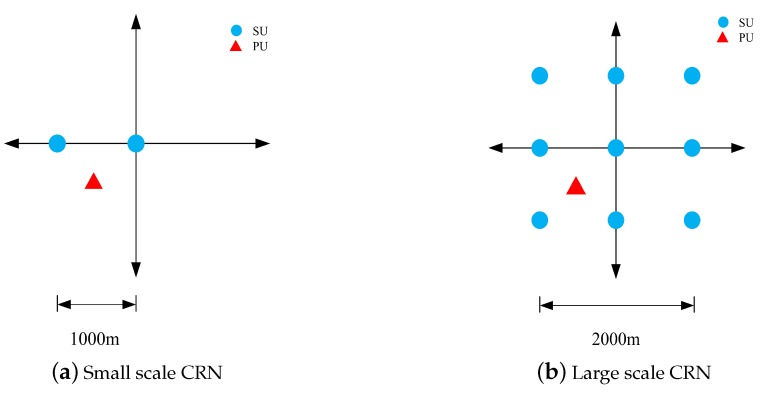
Layout of the cognitive radio networks (CRN).

In this section, the performance of the proposed hybrid AdaBoost classification algorithm is evaluated in computer simulations. We consider both a small scale CRN and a large scale CRN and the layout of a small scale CRN with two SUs participating in CSS is shown in Figure 5a, where the SU are located respectively at (−1, 0) km and (0, 0) km and the PU at (−0.6,−0.6) km. The large scale layout with nine SU is shown in Figure 5b, where all the SU are uniformly scattered within a 2.0 km × 2.0 km square area and the single PU is located at (−0.6,−0.6) km.

The PU signal bandwidth *W* is fixed as 5 MHz, the sensing duration τ is set as 100 μs, and the path-loss exponent ρ is chosen as 4. The shadow fading and the multi-path fading components are set as normal random variables with $E\left[\psi_{n}\right]=E\left[v_{n}\right]=0\;\mathrm{dB},\mathrm{Var}\left[\psi_{n}\right]=1\;\mathrm{dB}$, and $\mathrm{Var}[v_{n}]=5\;\mathrm{dB}$. In addition, the number of training samples *L* is 1000.

### 6.1. Prediction Error for Different Classifiers

**Figure 6:**
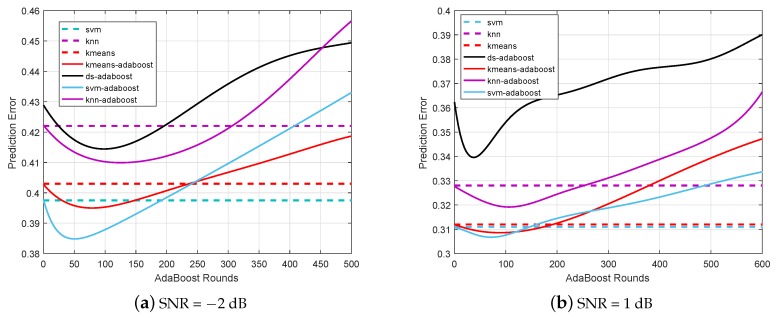
Prediction error when two secondary users (SUs) participate in cooperative spectrum sensing (CSS). K-nearest neighbors (KNN), decision stumps (DS).

**Figure 7:**
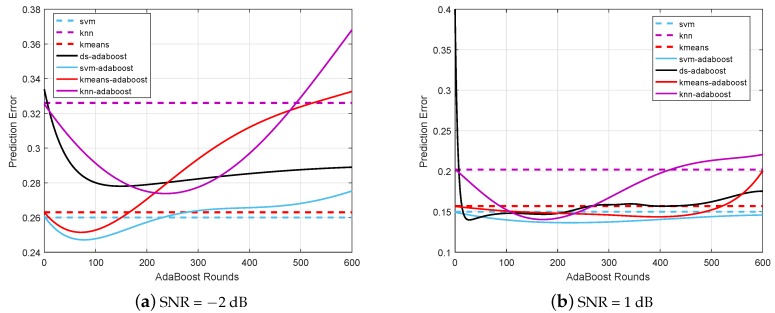
Prediction error when nine SUs participate in CSS.

The prediction errors of different ML technique based CSS algorithms for different sets of cooperative SUs are evaluated and compared in Figure 6 and Figure 7, respectively. It is shown that the prediction error of the proposed hybrid AdaBoost (e.g., SVM-AdaBoost, K-Means-AdaBoost, and KNN-AdaBoost) and the conventional DS-AdaBoost algorithms deteriorate when the number of AdaBoost rounds increases. Although the prediction errors of SVM, KNN, and K-Means algorithms remain consistent, they are inferior to the hybrid AdaBoost algorithms respectively. The prediction error performance is closely related to the number of SUs participating in CSS, since it is shown to be unacceptably deteriorating when the number of the cooperative SUs is as small as two. However, it is apparent that for the number of AdaBoost rounds being approximately 50, the SVM-AdaBoost algorithm achieves the best performance among all the algorithms, regardless of the number of SUs participating in the CSS.

It is important to note that the DS-AdaBoost algorithm and the proposed hybrid AdaBoost algorithms both encounter the overfitting problem when the number of AdaBoost rounds is unnecessarily increased, which makes the prediction error increase. In particular, the overfitting point of both the DS-AdaBoost and the hybrid AdaBoost are approximately around 100 rounds. Through this case, it is suitable to choose the number of iterations less than 100 in hybrid AdaBoost. It is verified that the SVM-AdaBoost algorithm achieves the lowest prediction error among all hybrid AdaBoost algorithms, outperforming all the other algorithms investigated in this paper.

### 6.2. Detection Probability for Different Classifiers

**Figure 8:**
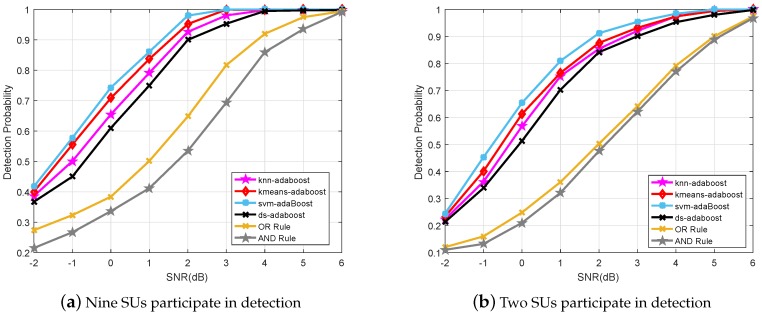
Detection probability with desired false alarm probability 10% and multiple SUs in CSS.

In addition to the prediction error, we also compare the detection probability performance of the conventional CSS schemes, e.g., hard decision fusion based AND and OR methods, and the hyrbid AdaBoost classifiers based CSS algorithms, as shown in Figure 8. The results are obtained, with 10-fold cross-validation, for a desired false alarm probability of 10% when nine and two SUs participate in CSS, respectively. Simulation results show that all the ML classifiers based CSS schemes studied in this paper outperform the traditional logical based CSS methods. Moreover, the proposed SVM-AdaBoost scheme performs the best among all the CSS schemes.

### 6.3. Training Duration and Prediction Duration

The training duration for different classifiers with different numbers of training samples are shown in Table 1. The training duration is reasonably increased with the increase of the number of training samples. The DS-AdaBoost shows the longest training duration (5.6157 s for 1000 training samples) among all these algorithms, whereas the proposed hybrid AdaBoost algorithms take relatively lower training durations. The proposed hybrid AdaBoost algorithms take less training time than the DS-AdaBoost algorithm, due to the high classification accuracy of the strong classifier that replaces the role of some DS in DS-AdaBoost and accelerates the training processing. The K-means classifier has the capability of detecting the spectrum availability more agilely in comparison to other ML classifiers. The times taken for deciding the spectrum availability for different classifiers with different numbers of test samples are shown in Table 2. It is clear to observe that for the same test samples readily given, the prediction durations of all classifiers increase more or less with the increase of the number of test samples and the KNN algorithm takes the longest time for determining the spectrum availability. In Table 2, it is also easy to notice that the SVM-AdaBoost algorithm takes the lowest time and the KNN-AdaBoost takes the longest time among the proposed hybrid AdaBoost algorithms when the number of test samples is the same.

## 7. Conclusions

In this paper, hybrid AdaBoost classification algorithms are proposed on the basis of different sub-classifiers, composed of a strong ML classifier and multiple weak DS, for cooperative spectrum sensing in cognitive radio networks. During spectrum sensing operation, the energy vectors collected from the cooperative SUs are used as the feature vectors to determine the spectrum availability for the SU. The proposed hybrid AdaBoost algorithms achieve lower prediction errors than the conventional DS based AdaBoost algorithm. Meanwhile, among various hybrid AdaBoost algorithms, SVM-AdaBoost exhibits the best performance in terms of prediction error and detection probability, in comparison with other hybrid AdaBoost algorithms and with conventional cooperative spectrum sensing methods. With the salient performance and technical merits, the SVM-AdaBoost algorithm may serve as a practical machine learning based solution for cooperative spectrum sensing.

## Figures and Tables

**Table 1:** Training duration for different classifiers.

Classification Method	Number of Training Samples					
	100	200	300	400	500	1000
SVM	0.0203	0.0254	0.0320	0.0391	0.1207	0.1466
K-means	0.0145	0.0168	0.0171	0.0178	0.0205	0.0542
KNN	0.0421	0.0441	0.0466	0.0502	0.0543	0.0967
DS-AdaBoost	5.0670	5.1078	5.1955	5.2169	5.3562	5.6157
SVM-AdaBoost	4.9518	4.9538	4.9640	4.9767	4.9857	5.0912
K-means-AdaBoost	4.9343	4.9423	4.9478	4.9498	4.9585	5.0053
KNN-AdaBoost	5.1258	5.1299	5.1325	5.1386	5.1756	5.2287

**Table 2:** Prediction duration for different classifiers.

Classification Method	Number of Test Samples					
	100	200	300	400	500	1000
SVM	$1.156\times 10^{-3}$	$1.069\times 10^{-3}$	$1.219\times 10^{-3}$	$1.267\times 10^{-3}$	$1.752\times 10^{-3}$	$3.146\times 10^{-3}$
K-means	$2.12\times 10^{-2}$	$2.12\times 10^{-2}$	$2.12\times 10^{-2}$	$2.13\times 10^{-2}$	$2.13\times 10^{-2}$	$2.15\times 10^{-2}$
KNN	$1.75\times 10^{-1}$	$1.76\times 10^{-1}$	$1.77\times 10^{-1}$	$1.78\times 10^{-1}$	$1.82\times 10^{-1}$	$2.31\times 10^{-1}$
DS-AdaBoost	$4.94\times 10^{-2}$	$8.50\times 10^{-2}$	$9.74\times 10^{-2}$	$1.45\times 10^{-1}$	$2.19\times 10^{-1}$	$3.35\times 10^{-1}$
SVM-AdaBoost	$1.65\times 10^{-2}$	$1.65\times 10^{-2}$	$1.66\times 10^{-2}$	$1.66\times 10^{-2}$	$1.68\times 10^{-2}$	$1.7\times 10^{-2}$
K-means-AdaBoost	$2.78\times 10^{-2}$	$2.78\times 10^{-2}$	$2.78\times 10^{-2}$	$2.80\times 10^{-2}$	$2.80\times 10^{-2}$	$2.83\times 10^{-2}$
KNN-AdaBoost	$1.76\times 10^{-1}$	$1.76\times 10^{-1}$	$1.77\times 10^{-1}$	$1.79\times 10^{-1}$	$1.83\times 10^{-1}$	$2.32\times 10^{-1}$
